# The relationship between family function and personality traits with general self-efficacy (parallel samples studies)

**DOI:** 10.1186/s40359-020-00462-w

**Published:** 2020-08-27

**Authors:** Ali Zakiei, Hosna Vafapoor, Mostafa Alikhani, Vahid Farnia, Farnaz Radmehr

**Affiliations:** 1 Sleep Disorders Research Center, Kermanshah University of Medical Sciences, Kermanshah, Iran; 2 Department of Psychology, Faculty of Social Sciences, Razi University, Kermanshah, Iran; 3 Substance Abuse Prevention Research Center, Health Institute, Kermanshah University of Medical Sciences, Kermanshah, Iran

**Keywords:** General self-efficacy, Personality traits, Family functions

## Abstract

**Background:**

General Self-efficacy is a key variable in clinical, educational, social, developmental, health and personality psychology that can affect the outcomes of people’s lives. The present study aimed to investigate the relationship between family functions and personality traits with general self-efficacy among university students and the general population.

**Methods:**

To conduct this two-part study, the first part was carried out on a sample of 500 students, and in the second part the study was repeated on a larger sample consisting of 1000 participants from the general population data were collected from the General Self-Efficacy Scale (GSE), Family Assessment Device (FAD), and the Revised NEO Personality Inventory (NEO PI-R). The analysis was performed using Pearson’s correlation coefficient, Fisher’s z test and regression analysis.

**Results:**

The results of the present study revealed that all the subscales of family functions and all personality traits are significantly related to general self-efficacy among university students and general population (*p* < 0.001). But in the general population, there was no significant correlation between openness to experience with general self-efficacy) (*p* > 0.05). Furthermore, the results of regression analysis showed subscales of family functions and all personality traits together can predict 27 and 35% of the variance in general self-efficacy among university students and the general population, respectively.

**Conclusion:**

Personality traits play a role in predicting general self-efficacy, but the personality trait of conscientiousness plays a greater role than other personality traits and also compared to family functioning, personality traits play a greater role in predicting general self-efficacy.

## Background

General self-efficacy is one of the interesting psychological issues for researchers. So that some consider self-efficacy as an important precondition for self-management in the process [1]. Some researchers have shown that general self-efficacy is an important predicting factor for academic achievement at various levels of education [2]. Furthermore, self-efficacy is one of the factors influencing health promotion, for example, it has an important role in reducing high-risk behaviors leading to HIV [3–5]. Moreover, it is also associated with behaviors such as oral health behaviors and smoking [6, 7]. On the other hand, its relationship with depression, anxiety, and other mental health indicators have also been confirmed [8–10].

In the psychology literature, general self-efficacy is considered as a motivational construct and has been defined as a person’s belief in his abilities and competencies for the success of a particular assignment [11, 12]. Self-efficacy beliefs mean confidence in our ability to organize, manage, and control life situations [13]. Bandura believes that the origins of self-efficacy are from early family experiences [13].

Family function or efficiency is a collaborative effort to the establishment and maintain the family balance. A family with an optimal function is an open system whose members are emotionally interconnected, but members have nevertheless been encouraged to expand their identities. Such a family is full of love and every family member is accepted unconditionally. As a result of this acceptance, the family can resolve conflicts and willingly respond to the request for help from members [14].

The family function in essence refers to the systemic characteristics of the family. In other words, family function means the ability of the family to protect the entire family system to keep pace with changes in life, to resolve conflicts, to reach union among members and success in discipline, compliance with the boundaries between individuals and the enforcement of the rules and principles governing the family institution. One of the important models in the field of family function is McMaster’s family pattern. This pattern was introduced by Epstein, Baldwin & Bishop in 1983 at McMaster University [15].

Family function evaluation according to this model is based on a system approach. The model utilizes a general systems theory approach in an attempt to describe the structure, organization, and transactional patterns of the family unit. The basic principles of this model include: the relationship between parts and components of the family with each other, the incomprehensibility of a component separate from other components of the family, the important role of structure and Interactive patterns of family in determining and shaping the behavior of family members [16]. Therefore, in this study, considering the comprehensiveness of this model, it was used.

Personality marks the relatively stable individual difference in physical appearance, behavior and experience of humans over time [17].

One of the most prominent personality models is the model of the five big personality factors [18], which is the dominant approach for representing the human trait structure today [19].

The big five personality factors include: neuroticism, extraversion, agreeableness, conscientiousness, and openness to experience. Neuroticism refers to the vulnerability to emotional instability and self-consciousness. Openness to experience is characterized by the cognitive disposition to creativity and esthetics. Agreeableness and extraversion focus on the interpersonal relationship: Extraversion reflects the tendency to be gregarious, enthusiastic, assertive, and to seek excitement, whereas agreeableness refers to the tendency to be warm, kind, gentle, trusting, and reliable. Conscientiousness is understood as the tendency toward dutifulness and competence [20].

A review of previous studies shows that general self-efficacy and personality traits are strongly related. For example, the results of the study Judge & Ilies (2002) indicated that there was a negative correlation between general self-efficacy and neuroticism. Also, the results of this study showed that there were positive correlations between general self-efficacy and variables of extraversion, openness to experience, and conscientiousness [21]. The results of another study indicated that, besides the four traits mentioned by Judge and Ilies (2002), agreeableness and general self-efficacy were significantly related, and neuroticism could negatively predict general self-efficacy [22]. Ebstrup et al. (2011) in their study, confirmed the findings of Judge and Ilies (2002) and showed that self-efficacy beliefs significantly predict the relationship between the four factors of neuroticism, extraversion, openness to experience, and conscientiousness [22].

For researchers, there is a question that self-efficacy is more influenced by personality traits or family factors? So far, no research has responded to this question. Responding to this question may help to a better understanding of this concept. On the other hand, it seems that to answer this important question, we need to study simultaneously (parallel), so that we can compare the results with each other.

A more comprehensive understanding of the interactions between family function and personality traits with self-efficacy seems necessary. There are claims in this regard that make the problem complicated and confusing. For example, some people believe that the impact of the life important persons (for example parents) in development of self - efficacy has been declared previously [23, 24]. On the other hand, some researchers have confirmed that there is a relationship between self-efficacy and personality traits [22]. Their results showed that there is a relationship between all five big personality factors and self-efficacy [22].

## Rationale and objectives

General Self-efficacy is a key variable in clinical, educational, social, growth, health and personality psychology that can affect the outcomes of people’s lives.

General self-efficacy plays a key role in academic achievement. The results of studies conducted on 23 countries worldwide indicated the fact that general self-efficacy and academic achievement have a positive relationship [25].

Considering the importance of self-efficacy in health, improving the performance and success of individuals in life and education, carrying out studies on self-efficacy among university students and other people seems necessary. Also, paying attention to the associated factors with general self-efficacy can help us to a better understanding of the matter of this concept. Therefore, the present study aimed to investigate the relationship between family functions and personality traits with general self-efficacy among university students and the general population.

In previous studies conducted in a statistical sample, personality and family variables were not investigated simultaneously. On the other hand, comparing correlations and impact coefficients in two statistically different age samples can help us better understand general self-efficacy.

Considering the importance of self-efficacy in daily life, and to allow for comparing the results between two different age groups, we have selected two distinct samples which made it possible to investigate and evaluate the relationship between family function and personality traits with general self-efficacy.

Also, this study was conducted to investigate the role of family functioning and personality traits in predicting general self-efficacy and we were looking for an answer to the question that which components of family functioning and personality traits play a greater role in predicting general self-efficacy?

## Methods

### Study design and participants

This correlational study included two parts. In the first part, the statistical population included all students at Kermanshah University of Medical Sciences in 2015. The age range of the participants was 18–27 years.

Sampling method in the first study, the random stratified sampling method was that each faculty was considered as a community category and within each category sampling was done by simple random sampling. Random selection of individuals in each faculty was done in such a way that first by throwing the dice and the number 4 came, the researcher selected the students who entered the faculty according to the multiple of 4 from the entrance of the university, and if desired and qualified Being entered into the study according to the inclusion and exclusion criteria of the research. This process continued until the sample size was completed in each faculty. Thus, 500 people were selected as a sample from different faculties.

To determine the sample size, the Cochran’s sample size formula, depicted below, was used. Cochran’s Formula for estimating the size of the sample: $n=\frac{Z_{1-\frac{\alpha }{2}}P\left(1-P\right)}{d^2}$. (z = 1.96, d = 0.05, *p* = 0.5). Based on this formula, the size of the sample was determined 350. Considering the possibility of a drop of participants and to decrease the second type error, the size of the sample for the study was determined 500.

Participants were asked to respond to the questionnaires. Detailed explanations on how to complete the questionnaires were provided by the researchers, and the participants were requested to ask for more clarification in case of encountering problems filling out the questionnaires. Further, the questionnaires were completed individually in the presence of the researchers. Finally, the questionnaires were collected and the obtained data were analyzed.

In the second part of the study, the statistical population included all individuals aged 20 to 60, have been living in Kermanshah City (Iran) for at least 5 years before the study and did not have the conditions for entering the student group.

For the second sample, 1000 participants were selected. Because of the lack of a list of the statistical population and the possibility to utilize random sampling (the most appropriate method in this study) two-stage cluster sampling was recognized. In this method, the city was first divided into six regions (the criterion was the municipal areas). After that, each area was divided into clusters and from each cluster, the samples were randomly selected.

Also, the random selection of individuals in each cluster was performed in this way that the researcher first stood on the main street of that area, and selected the passengers whose order of passing was according to the multiple of 4, then if they were willing, were included in the study according to the criteria of inclusion and exclusion.

Individuals volunteered to participate in this study and signed an informed consent form. They were also assured of the confidentiality of the information contained in the questionnaires. This study was registered in the Research Center of Kermanshah University of Medical Sciences and approved by the local ethics committee.

### Instruments

The data collection instruments were similar in both studies, and three questionnaires were used: the general self-efficacy scale (GSE), family assessment device (FAD), and the revised NEO personality inventory (NEO PI-R).

#### The general self-efficacy scale

This 10-item psychometric scale with multiple choice questions (MCQ) was developed by Schwarzer, Jerusalem and Romek [26]. Moreover, each question can have a score of 1 to 4 (1 = not at all true, 2 = hardly true, 3 = moderately true, 4 = exactly true), and the total score of the questionnaire range from 10 to 40. Its Cronbach’s alpha and internal consistency coefficient have been reported to be .81–.91 [27]. This scale was normalized by Rajabi [28] in Iran (α = 0.82). In the present study, Cronbach’s alpha was 0.88 for the first sample and 0.91 for the second one.

#### The family assessment device (FAD)

This 60-item scale was developed based on the McMaster model of family functions, and it measures the structural, occupational, and interactive characteristics of families. This model evaluates six dimensions of family function: 1- Problem-solving 2- Communication 3- Roles 4- Affective involvement 5- Behavior Control 6- Affective Response [29].

The response option for each statement was 1 = strongly agree, 2 = agree, 3 = disagree, and 4 = strongly disagree. After recoding positively oriented items, item scores were summed to obtain a total score, which could range from 60 to 240, with higher scores representing better functioning [30]. The scoring is based on the Likert spectrum, lower scores indicate healthier functioning. Furthermore, the reliability of the scale was reported .72–.92 [30]. The reliability and validity of this scale have been confirmed in various studies [31]. The FAD shows good reliability and validity, with a Cronbach’s alpha coefficient between 0.78 and 0.86 [32]. Also, Zadehmohamadi and Malekkhosravi (2006), by Cronbach’s alpha, the reliability coefficient for the total questionnaire 0.71 and the sub-scales, Problem-solving 0.72, Communication 0.70, Roles 0.71, Affective Involvement 0.73, Behavior Control 0.66, Affective Response 0.71 are reported [33]. In the present study, the Cronbach’s alpha coefficient was 0.86 for the first sample and 0.92 for the second one.

#### The revised NEO personality inventory

In the current study, the short form of the NEO Five Factor personality inventory was applied. In 1989, Costa and McCrae have designed the short form of the five factor inventory for measuring the five main personality traits, which included conscientiousness, neuroticism, openness to experience, agreeableness, and extraversion. This questionnaire includes 60 items and only measures the five main personality traits. The procedure for developing this scale was such that using factor analysis, 12 questions with the highest factor weight for all five factors were selected to form the short form of the inventory [34]. The short form of the NEO Five Factor Inventory has been translated into many languages and has been validated many times. Roshanchsly et al. [35] and Pakdaman et al. [36], validated this inventory. The responses to the questions in this inventory are based on a five-option Likert spectrum ranging from “completely disagree = 5” to “completely agree = 1”. In the current study, the Cronbach’s Alpha coefficient for the questionnaire on the first sample set was 0.79, while it was 0.66 for the second sample of the study. Furthermore, in the present study, the Cronbach’s Alpha coefficients for neuroticism (N), extraversion (E), agreeableness (A), conscientiousness (C), and openness to experience (O) were 0.71, 0.82, 0.86, 0.78, 0.75, *respectively*, for the students sample, and the Cronbach’s alpha coefficients for neuroticism, extraversion, agreeableness, conscientiousness, and openness to experience were 0.69, 0.80, 0.88, 0.71, and 0.70, respectively, for the general population. In a study, Cronbach Alpha coefficients for the scales were 0.84, 0.79, 0.74, 0.72, and 0.82 for N, E, O, A, and C respectively [37].

### Data analysis

Descriptive statistics were used as mean and standard deviation. The One-Sample Kolmogorov-Smirnov test was performed to evaluate the normal distribution of depression scores, personality traits, general self-efficacy, and family function. Pearson correlation coefficient test to investigate the relationship between personality traits and family function with general self-efficacy, Fisher’s z test to compare correlation coefficients and linear regression model to predict general self-efficacy (dependent variable) based on personality traits and family function (independents variable) were used. All analyses were performed using SPSS21 statistical software (JB39397R39KFC9) at a significant level of 5%.

## Results

The mean age in the students group (21.78 ± 2.28) was lower than the general population (33.49 ± 10.28) (Student’s t-test =25.18, < 0.001). In both groups, the majority of people were female, with a percentage of 58% in the student group and 55.8% in the general population, respectively. In Table 1, the mean and standard deviations of the variables studied are in the whole sample.

**Table 1:** Mean values of general Self-efficacy, Family Function, and Personality Traits

Variable		Study 1 (University Students)		Study 2 (The General Population)		Whole sample	
		Mean	SD	Mean	SD	Mean	SD
general Self-efficacy		29.31	4.88	29.39	5.55	29.35	5.23
Family Function	Communication	14.24	3.001	20.65	2.85	17.44	2.95
	Roles	20.68	3.53	24.17	3.41	22.42	3.55
	Affective Response	19.75	4.83	18.69	3.08	19.22	3.98
	Problem solving	11.16	2.94	18.65	2.77	14.91	2.89
	Affective Involvement	16.08	3.22	24.83	4.62	20.45	3.92
	Behavior Control	20.06	3.46	24.80	3.31	22.43	3.45
	General Functioning	26.99	5.25	37.82	5.12	32.40	5.22
	Total (FAD)	129.45	20.78	169.86	20.52	149.66	20.65
Personality Traits	Neuroticism	31.84	5.68	20.60	6.55	26.22	6.18
	Extraversion	40.24	5.98	28.63	5.78	34.59	5.88
	Agreeableness	40.55	5.43	28.50	5.62	34.53	5.89
	Conscientiousness	41.28	6.48	31.54	6.37	36.42	6.49
	Openness to Experience	38.01	4.33	29.38	4.42	33.69	4.56

To examine the relationship between family functions and personality traits with GSE in both groups selected for the study, the Pearson’s correlation coefficient was employed.

The results of Table 2 showed that in both studies, there was a significant negative relationship between all family subscales and self-efficacy. This means that increasing self-efficacy is possible by reducing each of the subscales and the overall family function score.

**Table 2:** Correlation coefficients between Family functions and Personality Traits with general Self-efficacy

		Study 1 (University Students)		Study 2 (The General Population)		Fisher’s z test	
		r	P^a^	r	p^a^	Z	p^b^
Family Functioning	Communication	− 0.32	0.001	−0.40	0.001	1.68	0.093
	Roles	−0.21	0.001	−0.30	0.001	1.75	0.080
	Affective Response	−0.27	0.001	−0.32	0.001	1.00	0.317
	Problem solving	−0.26	0.001	−0.27	0.001	0.2	0.841
	Affective Involvement	−0.25	0.001	−0.33	0.001	1.59	0.111
	Behavior Control	−0.22	0.001	−0.30	0.001	1.56	0.118
	General Functioning	−0.35	0.001	−0.43	0.001	1.72	0.085
	Total (FAD)	−0.50	0.001	−0.42	0.001	1.85	0.064
Personality Traits	Neuroticism	−0.32	0.001	−0.37	0.001	1.03	0.303
	Extraversion	0.37	0.001	0.40	0.001	0.64	0.522
	Agreeableness	0.22	0.001	0.18	0.001	0.76	0.447
	Conscientiousness	0.43	0.001	0.50	0.001	1.63	0.103
	Openness to Experience	0.12	0.004	0.05	0.121	1.28	0.200

^a^ p = Obtained from the Pearson correlation coefficients

^b^ p = Obtained from the Fisher’s *z* test

In general, there was a significant negative relationship between total FAD and self-efficacy with a correlation coefficient of − 0.50 at the level of *P* < 0.001 in the first study and with a correlation of − 42 at − 0 P < 0.001 in the second study (lower scores in FAD questionnaire indicate healthier functioning). On the other hand, only in the first study, there was a significant positive relationship between the openness to experience personality trait and self-efficacy (*p* < 0.05). Therefore, only among students increased the openness to experience personality by increasing self-efficacy. Also, the results showed that in the first and second studies, there was a significant positive relationship between the personality traits of extraversion, agreeableness, and conscientiousness with self-efficacy. In that sense, that with the increase of each personality traits (extraversion, agreeableness and conscientiousness), the level of self-efficacy increased. However, in both studies, the neuroticism personality trait with self-efficacy had a significant negative relationship with *p* < 0.001 and this personality trait had a reverse effect on self-efficacy. Also, the results of Fisher test showed that there was no significant difference between the two groups in the correlations. Therefore, it can be said that the relationship between personality traits and self-efficacy was not influenced by the groups we studied.

In order to predict general self-efficacy based on family functions and personality traits, regression analysis was utilized (Table 3).

**Table 3:** Results of regression analysis with Family functions and Personality Traits as predictors and Self-Efficacy as dependent variable

Study	Summary	Predictor Variable	B	β	t	p^**^
Study 1 (University Students)	R = 0.52 R^2^ = 0.27 F = 17.02 p^*^ = 0.001	Communication	−0.25	−0.15	2.78	0.006
		Roles	−0.11	−0.08	1.39	0.160
		Affective Response	− 0.08	−0.08	1.21	0.231
		Problem solving	−0.12	−0.07	1.25	0.211
		Affective Involvement	−0.10	−0.07	1.20	0.232
		Behavior Control	−0.07	−0.05	0.94	0.353
		General Functioning	−0.17	−0.18	2.86	0.004
		Neuroticism	−0.12	−0.14	3.08	0.002
		Extraversion	0.11	0.14	2.86	0.004
		Agreeableness	0.11	0.12	2.63	0.009
		Conscientiousness	0.23	0.30	6.16	0.001
		Openness to experience	0.01	0.01	0.31	0.762
Study 2 (The General Population)	R = 0.59 R^2^ = 0.35 F = 43.85 p^*^ = 0.001	Communication	− 0.29	−0.16	3.86	0.001
		Roles	−0.01	−0.07	0.17	0.862
		Affective Response	− 0.03	−0.02	0.48	0.634
		Problem solving	− 0.21	−0.11	2.73	0.006
		Affective Involvement	−0.01	−0.001	0.08	0.934
		Behavior Control	−0.01	−0.01	0.18	0.861
		General Functioning	−0.23	−0.12	4.62	0.001
		Neuroticism	−0.10	−0.12	3.68	0.001
		Extraversion	0.10	0.11	2.86	0.004
		Agreeableness	0.09	0.09	2.95	0.003
		Conscientiousness	0.26	0.32	9.37	0.001

R = multiple correlation coefficient

R^2^ = the coefficient of determination in regression analyses

p^*^ = Levels of significance of the Regression model

B = Unstandardized coefficient and slope of the regression line

β = Standardized coefficient in regression analyses

p^**^ = Levels of significance of Beta coefficients

The results of the regression analysis of the first study showed that the subscales of family functions and personality traits together can predict 27% of the variance in general self-efficacy. Among the subscales of family functioning, the subscale of communication (− 0.15) and general functioning (− 0.18) could predict general self-efficacy. In addition, among the personality traits, neuroticism (− 0.14), extraversion (0.14), agreeableness (0.12) and conscientiousness (0.30) were able to predict general self-efficacy. These results showed that in the first study, the relationship between family function and personality traits of neuroticism had a significant role in decreasing self-efficacy, and personality traits of responsibility, extroversion and conscientiousness had a significant role in increasing self-efficacy.

Additionally, the results of enter method regression analysis indicated that among the predicting variables, conscientiousness can predict general self-efficacy the most. Also, in a separate regression analysis, in which the total score of family functions was entered into the equation, the results indicated that it could predict general self-efficacy with an effect size of − 0.15. Therefore, according to the results of analysis in the first study, it can be argued that conscientiousness plays the most prominent role in predicting general self-efficacy compared to all the other variables.

Furthermore, the results of the regression analysis in the second study revealed that the subscales of family functions and personality traits together could predict 35% of the variance in general self-efficacy. Among the subscales of family functioning, the subscales of communication, problem solving and general functioning were able to predict general self-efficacy. Additionally, neuroticism, extraversion, agreeableness and conscientiousness were able to predict general self-efficacy. The regression results also showed that in the second study, the relationship between subscales, Communication, Problem solving and General Functioning as well as personality traits of neuroticism have a significant and decreasing role in self-efficacy and also personality traits of responsibility, extroversion and conscientiousness have a significant and increasing role in self-efficacy.

Additionally, the results of enter method regression analysis indicated that among the predicting variables, conscientiousness can predict general self-efficacy the most. Also, in a separate regression analysis, in which the total score of family functions was entered into the equation, the results indicated that it could predict general self-efficacy with an effect size of − 0.20. So, according to the results of the analysis in the second study, it can be argued that conscientiousness played the most prominent role in predicting general self-efficacy compared to all the other variables.

Given that the status of the age variable in the second sample (the general publicatin) was different from that in the sample consisting of university students, it was decided that the role of age is to be controlled in the relationship between family function and personality traits and general self-efficacy (Table 4).

**Table 4:** The Correlation of Personality Traits and Family functions with general self-efficacy and Standardized Regression Coefficients for prediction general self-efficacy in Separate Age Groups in the Second Study

Age		lower30 (***n*** = 445)	30–50 (***n*** = 489)	More 50 (***n*** = 66)	Total
Neuroticism	r	− 0.36^b^	− 0.38^b^	− 0.34^b^	− 0.36^b^
	β	−0.04	−0.21^b^	0.03	
Extroversion	r	0.49^b^	0.33^b^	0.12	0.41^b^
	β	0.14^a^	0.09	–	
Conscientiousness	r	0.57^b^	0.45^b^	0.34^b^	0.49^b^
	β	0.32^b^	0.39^b^	0.17	
Agreeableness	r	0.21^b^	0.10^a^	0.41^b^	0.19^b^
	β	−0.03	0.20^b^	0.44^a^	
Openness to Experience	r	0.04	0.08	0.34^b^	0.08
	β	–	–	0.13	
Problem Solving	r	− 0.40^b^	− 0.18^a^	− 0.22^b^	−0.29^b^
	β	− 0.14^a^	−0.02	−0.05	
Communication	r	− 0.51^b^	− 0.30^b^	−0.32^b^	−0.38^b^
	β	− 0.26^b^	− 0.12	−0.05	
Roles	r	− 0.39^b^	− 0.20^b^	− 0.20	− 0.28^b^
	β	0.04	−0.07	–	
Affective Response	r	−0.43^b^	− 0.20^b^	−0.55^b^	−0.33^b^
	β	0.09	− 0.17^b^	−0.42^b^	
Affective Involvement	r	− 0.38^b^	− 0.26^b^	−0.09	−0.31^b^
	β	−0.21^b^	0.21^b^	–	
Behavior Control	r	−0.36^b^	− 0.25^b^	−0.08	−0.30^b^
	β	−0.07	−0.08	–	
General Functioning	r	−0.57^b^	− 0.27^b^	−0.07	−0.44^b^
	β	−0.34^b^	−0.07	–	

^a^. Significant at the 0.05 level (2-tailed)

^b^. Significant at the 0.01 level (2-tailed)

*β* Standardized coefficient in regression analyses.

The results of the analysis showed that, in the group aged under 30 years old, family functions and personality traits together were able to predict 47% of general self-efficacy, while this rate was equal to 29 and 53% in the 30–50 age range and the age group over 50 years old, respectively. Therefore, the highest impact of personality traits and family function on self-efficacy was in the age group above 50 and under the age group of 30 years.

The results depicted in Table 4 show that by controlling the age variable (split-half correlation), a significant relationship was found between all personality traits, except for openness to experience, and family functioning. Also, in the group aged less than 30 years old, the highest effect sizes belonged to general family functions and conscientiousness, respectively. Moreover, in the 30–50 age group, the highest effect size belonged to conscientiousness. In other words, conscientiousness played a prominent role in predicting general self-efficacy. As for the third age range, i.e. older than 50 years, agreeableness played a significant role in predicting general self-efficacy. Comparing the mean scores of general self-efficacy of the three age groups showed that there was no significant difference between these three groups with regards to general self-efficacy.

For controlling the role of age in predicting self-efficacy in the second sample, multiple regression analysis was used, and the results are presented in Table 5. The results of the analysis showed that age had a moderating role in the relationship between variables.

**Table 5:** Results of regression analysis with the independent variables in the second sample for controlling of age

Study	Summary	Predictor Variable	B	β	t	P^**^
Study 2 (The General Population)	R = 0.59 R^2^ = 0.35 F = 39.41 P^*^ = 0.001	Communication	0.29	0.16	3.78	0.001
		Roles	−0.03	−0.02	0.44	0.65
		Affective Response	− 0.02	−0.01	0.28	0.78
		Problem solving	− 0.18	−0.10	2.36	0.02
		Affective Involvement	−0.02	−0.02	0.48	0.68
		Behavior Control	0.02	0.01	0.38	0.70
		General Functioning	0.21	0.20	4.11	0.001
		Neuroticism	−0.09	−0.11	3.29	0.001
		Extraversion	0.10	0.11	2.98	0.003
		Agreeableness	−0.07	−0.08	2.44	0.01
		Conscientiousness	0.27	0.33	9.66	0.001
		Openness to Experience	−0.12	−.10	3.49	0.001

## Discussion

Our findings revealed that there was a relationship between family functions and general self-efficacy in both studies. This means that the healthier functioning of the family, the higher the general self-efficacy of its members. These results were in line with the results of studies conducted by Hall [38]; Caprara et al. [39]; Hoeltje et al. [40]; Lotfinia et al. [41].

To explain the relationship between family functions and general self-efficacy, it is noteworthy that a healthy family with optimum functioning is supported by its members, and general self-efficacy can be strengthened by social support [42]. Additionally, families with optimum functioning boast parenting styles that can nurture self-efficacy beliefs [12]. Moreover, the most important explanation for this part of the results is Bandura’s perspective based on social learning the family is regarded as one of the crucial sources for imitation and mimicking of general self-efficacy, and parental behaviors and lifestyles are effective patterns for nurturing general self-efficacy [12].

The children of from families with proper functioning are free to have their say and are provided with the opportunity to express their thoughts on various issues and make suggestions when necessary. As a result, this feeling is bred that they will be able to find suitable solutions for problems, believe in their abilities, and experience higher efficacy. Also, children can express their strengths and weaknesses without any fear in dialogue and interaction-inducing atmosphere, resulting in strengthening one’s beliefs in their strengths, their ability to find solutions to their weaknesses, and their general self-efficacy.

The results of the present study suggested that the general family functions had the strongest correlation with general self-efficacy among all the components of family functions in both groups. The results also showed that there was a relationship between all personality traits and general self-efficacy in the first study, while in the second one, four personality traits and general self-efficacy were correlated, and no relationship was observed between openness to experience and general self-efficacy. In addition, the results of the present study indicated that conscientiousness had the strongest correlation with general self-efficacy in both groups.

Furthermore, the results of studies carried out by Judge and Ilies (2002), Caprara et al. (2005), Stroble et al. (2011),, Gerhardt et al. (2007), and McGeown et al. (2014) indicated that personality traits and general self-efficacy were correlated, which is consistent with the results of the present study [21, 39, 43–45]. According to the results of the present study, it can be expressed that the higher neuroticism trait is accompanied with the lower general self-efficacy. Additionally, it can be stated that the more the features of extraversion, agreeableness, conscientiousness, and openness to experience are increased, the higher increased the general self-efficacy will be, and vice versa. Judge et al. (2007) and Saleem et al. (2011) concluded that self-efficacy was correlated with personality traits, especially neuroticism and extraversion [46, 47]. Also, the results of a study on teachers showed that self-efficacy was positively correlated with conscientiousness, openness to experience, and agreeableness, whereas no relationship was observed between neuroticism and self-efficacy [48]. In this study, agreeableness and general self-efficacy were strongly related, and the reasons for the inconsistency of the results could be the sample size and the instrument employed for measuring general self-efficacy.

Also people with neuroticism display incompatibility when faced with tough conditions, and so they are not capable of managing and controlling situations, and have low self-esteem. Therefore, general self-efficacy is also low [48]. On the other hand, extroverts seek the support of others when faced with tough conditions because of their high flexibility [39, 49]. They also receive the encouragement of others because of higher sociability, leading to placing more emphasis on one’s capabilities and competency [50]. Besides, extroverts tend to express their ideas and feelings, which is deemed a great source for general self-efficacy [12].

To better explain the relationship between general self-efficacy and openness to experience, it can be stated that one with this personality trait will be interested in experiencing new and unfamiliar things [51]. That is why they feel that they can handle academic tasks and assignments and feel bound to carry out their tasks, increasing their efforts because they believe in themselves which will help them reach general self-efficacy. Moreover, those who are open to experience tend to meet challenging situations and get less disappointed when faced with unexpected situations; rather, they embrace such situations and display this characteristic most of the time [50].

The results of the present study showed that conscientiousness and general self-efficacy were correlated, which is consistent with the results of studies carried out by Colquitt & Simmering (1998) and Jones & Green (2001). It can be argued that it is not unexpected that one with more conscientiousness will have more general self-efficacy because they probably believe in their abilities and limitations and select realizable goals for themselves. People with conscientiousness are characterized as being diligent, feeling duty bound, not giving up in the face of obstacles, and being goal-oriented. They also perform their tasks carefully, which will help them achieve success, that will in turn result in the formation of feelings of competence and general self-efficacy [52, 53]. On the other hand, people with conscientiousness often work in groups, benefiting from the support of the group [49]. Therefore, the higher the level of one’s conscientiousness, the higher their general self-efficacy [21].

We suggested that there is a significant relationship between agreeableness and general self-efficacy among the students. However, in the general population (the second study), this relationship is not significant. To explain this finding of the study, we have to focus on the characteristics of the participants in the two samples since considering the age range of the students, agreeableness will be higher, and this trait is reduced when age increases.

The study results revealed that the subscales of family functions and personality traits together can predict between 27 and 35% of the general self-efficacy. These results explain that between 27 and 35% of the general self-efficacy is under the influence of family functions and personality traits, future researches can find other related variables.

Reciprocal determinism in social cognitive theory which was introduced by Bandura [54] explains the relationships among the environment, self, and behavior. Our study is matching this theory, because we investigated the family function and personality traits, so we considered the environment (Family), self (personality traits), and behavior (general self- efficacy).

Our results revealed that conscientiousness played the most prominent role in predicting general self-efficacy compared to all other variables.

Given the values of the regression coefficients in the regression analysis, it can be concluded that general self-efficacy is more personality-oriented and is highly influenced by personality traits, which is in line with theories stressing that general self-efficacy is personality-oriented. On the other hand, this finding can be intriguing for psychologists and behavioral science researchers.

We concluded that after controlling the age variable, a significant relationship was found between family functions and all personality traits, except for openness to experience, among the general population. In addition, in the group aged less than 30 years old, the general family functions and conscientiousness had the strongest correlations with general self-efficacy, respectively. This result is inconsistent with the general findings of the present study, indicating the importance of family in this age range. Moreover, in the 30–50 age range, the highest effect size belonged to conscientiousness. In other words, conscientiousness plays an important role in predicting general self-efficacy, which is in line with the overall findings of the present study. As for the third age group, i.e. those over the age of 50, agreeableness plays a considerable role in predicting general self-efficacy. The role of the age should be considered to determine which factor has more influence on self-efficacy, however, general self-efficacy is not influenced by age since the results of our study showed that there was no relationship between age and general self-efficacy.

As a result, Annesi (2007) reported that no change in general self-efficacy was seen by changing age [55].

The results of this study led us to the conclusion that self-efficacy is influenced by personality traits and these traits are more influenced by genetics and nature. It is suggested that in future research, the contribution of each of the nature and nurture factors in the formation of self-efficacy be determined.

### Limitations and strengths

Our study has several limitations. Due to the length of the research questionnaire questions, it may affect the accuracy of the participants’ answers. Therefore, it is suggested that shorter forms of these questionnaires be used in future research. Also, this research was conducted in one of the Kurdish cities of western Iran (Kermanshah), so the generalization of these results to other cultures and cities of Iran and the world should be cautious. Finally, the cross-sectional design was another limitation of the present study. It is recommended that longitudinal design be performed in the future. This study was conducted with a large sample conducted in western Iran. Also, participants were evaluated in the study sample by trained and experienced individuals.

## Conclusions

This study again highlights the role and importance of personality traits for researchers and psychologists. Since general self-efficacy plays an essential role in psychosocial health and human progression. This leads us to realize that the effect of personality traits can be considered through self-efficacy on psychosocial health and performance. However, other studies need to investigate the moderating role of self-efficacy in the relationship between personality traits and psychosocial health and performance.

## Supplementary information

**Additional file 1.**

**Additional file 2.**

**Additional file 3.**

## Data Availability

The datasets used and/or analyzed during the current study are available from the corresponding author on reasonable request.

## Abbreviations

GSE: General Self-Efficacy Scale
FAD: Family Assessment Device
NEO PI-R: Revised NEO Personality Inventory
MCQ: Multiple choice questions
N: Neuroticism
E: Extraversion
A: Agreeableness
C: Conscientiousness
O: Openness to experience
