# Supplementary material for: The relationship between family function and personality traits with general self-efficacy (parallel samples studies)
Source: BMC Psychol. 2020 Aug 27;8:88. doi: 10.1186/s40359-020-00462-w (PMC7457252; doi:10.1186/s40359-020-00462-w)
Supplement: Supplementary file 2 — Additional file 2. [file 40359_2020_462_MOESM2_ESM.pdf]

SECTION 4

This next section contains a number of statements about families. Please read each statement carefully, and decide how well it describes your own family. You should answer as to how you see your family.

For each statement there are four (4) possible responses:

Strongly Agree (SA)	Check **SA** if you feel that the statement describes your family very accurately.
Agree (A)	Check **A** if you feel that the statement describes your family for the most part.
Disagree (D)	Check **D** if you feel that the statement does not describe your family for the most part.
Strongly Disagree (SD)	Check **SD** if you feel that the statement does not describe your family at all.

Circle the response which best describes your feelings:

1. Planning family activities is difficult because we misunderstand each other.	**SA**	**A**	**D**	**SD**
2. We resolve most everyday problems around the house.	**SA**	**A**	**D**	**SD**
3. When someone is upset the others know why.	**SA**	**A**	**D**	**SD**
4. When you ask someone to do some-thing, you have to check that they did it.	**SA**	**A**	**D**	**SD**
5. If someone is in trouble, the others become too involved.	**SA**	**A**	**D**	**SD**
6. In times of crisis we can turn to each other for support.	**SA**	**A**	**D**	**SD**
7. We don't know what to do when an emergency comes up.	**SA**	**A**	**D**	**SD**
8. We sometimes run out of things that we need.	**SA**	**A**	**D**	**SD**
9. We are reluctant to show our affection for each other.	**SA**	**A**	**D**	**SD**

10. We make sure members meet their family responsibilities.	**SA**	**A**	**D**	**SD**
11. We cannot talk to each other about the sadness we feel.	**SA**	**A**	**D**	**SD**
12. We usually act on our decisions regarding problems.	**SA**	**A**	**D**	**SD**
13. You only get the interest of others when something is important to them.	**SA**	**A**	**D**	**SD**
14. You can't tell how a person is feeling from what they are saying.	**SA**	**A**	**D**	**SD**
15. Family tasks don't get spread around enough.	**SA**	**A**	**D**	**SD**
16. Individuals are accepted for what they are.	**SA**	**A**	**D**	**SD**
17. You can easily get away with breaking the rules.	**SA**	**A**	**D**	**SD**
18. People come right out and say things instead of hinting at them.	**SA**	**A**	**D**	**SD**
19. Some of us just don't respond emotionally.	**SA**	**A**	**D**	**SD**
20. We know what to do in an emergency.	**SA**	**A**	**D**	**SD**
21. We avoid discussing our fears and concerns.	**SA**	**A**	**D**	**SD**
22. It is difficult to talk to each other about tender feelings.	**SA**	**A**	**D**	**SD**
23. We have trouble meeting our bills.	**SA**	**A**	**D**	**SD**
24. After our family tries to solve a problem, we usually discuss whether it worked or not.	**SA**	**A**	**D**	**SD**
25. We are too self-centered.	**SA**	**A**	**D**	**SD**
26. We can express feelings to each other.	**SA**	**A**	**D**	**SD**
27. We have no clear expectations about toilet habits.	**SA**	**A**	**D**	**SD**
28. We do not show our love to each other.	**SA**	**A**	**D**	**SD**
29. We talk to people directly rather than through go-betweens.	**SA**	**A**	**D**	**SD**
30. Each of us has particular duties and responsibilities.	**SA**	**A**	**D**	**SD**
31. There are lots of bad feelings in the family.	**SA**	**A**	**D**	**SD**

32. We have rules about hitting people.	**SA**	**A**	**D**	**SD**
33. We get involved with each other only when something interests us.	**SA**	**A**	**D**	**SD**
34. There's little time to explore personal interests.	**SA**	**A**	**D**	**SD**
35. We often don't say what we mean.	**SA**	**A**	**D**	**SD**
36. We feel accepted for what we are.	**SA**	**A**	**D**	**SD**
37. We show interest in each other when we can get something out of it personally.	**SA**	**A**	**D**	**SD**
38. We resolve most emotional upsets that come up.	**SA**	**A**	**D**	**SD**
39. Tenderness takes second place to other things in our family.	**SA**	**A**	**D**	**SD**
40. We discuss who is to do household jobs.	**SA**	**A**	**D**	**SD**
41. Making decisions is a problem for our family.	**SA**	**A**	**D**	**SD**
42. Our family shows interest in each other only when they can get something out of it.	**SA**	**A**	**D**	**SD**
43. We are frank with each other.	**SA**	**A**	**D**	**SD**
44. We don't hold to any rules or standards.	**SA**	**A**	**D**	**SD**
45. If people are asked to do something, they need reminding.	**SA**	**A**	**D**	**SD**
46. We are able to make decisions about how to solve problems.	**SA**	**A**	**D**	**SD**
47. If the rules are broken, we don't know what to expect.	**SA**	**A**	**D**	**SD**
48. Anything goes in our family.	**SA**	**A**	**D**	**SD**
49. We express tenderness.	**SA**	**A**	**D**	**SD**
50.We confront problems involving feelings.	**SA**	**A**	**D**	**SD**
51. We don't get along well together.	**SA**	**A**	**D**	**SD**
52. We don't talk to each other when we are angry.	**SA**	**A**	**D**	**SD**
53. We are generally dissatisfied with the family duties assigned to us.	**SA**	**A**	**D**	**SD**

54. Even though we mean well, we intrude too much into each others lives.	**SA**	**A**	**D**	**SD**
55. There are rules about dangerous situations.	**SA**	**A**	**D**	**SD**
56. We confide in each other.	**SA**	**A**	**D**	**SD**
57. We cry openly.	**SA**	**A**	**D**	**SD**
58. We don't have reasonable transport.	**SA**	**A**	**D**	**SD**
59. When we don't like what someone has done, we tell them.	**SA**	**A**	**D**	**SD**
60. We try to think of different ways to solve problems.	**SA**	**A**	**D**	**SD**
