# Supplementary material for: The relationship between family function and personality traits with general self-efficacy (parallel samples studies)
Source: BMC Psychol. 2020 Aug 27;8:88. doi: 10.1186/s40359-020-00462-w (PMC7457252; doi:10.1186/s40359-020-00462-w)
Supplement: Supplementary file 3 — Additional file 3. [file 40359_2020_462_MOESM3_ESM.pdf]

NEO™-FFI-3

NEO Five-Factor Inventory-3

Item Booklet | **Form S–Adult**

SELF-REPORT

Paul T. Costa, Jr., PhD and Robert R. McCrae, PhD

Instructions

Write only where indicated in this Item Booklet. Carefully read all of the instructions before beginning. This questionnaire contains 60 statements. Read each statement carefully. For each statement, fill in the circle with the response that best represents your opinion. Make sure that your answer is in the correct box.

Fill in (SD) if you *strongly disagree* or the statement is definitely false.

Fill in (D) if you *disagree* or the statement is mostly false.

Fill in (N) if you are *neutral* on the statement, if you cannot decide, or if the statement is about equally true and false.

Fill in (A) if you *agree* or the statement is mostly true.

Fill in (SA) if you *strongly agree* or the statement is definitely true.

Note that the responses are numbered in ***rows***.

Example

First five responses from an individual who strongly disagrees with items 1, 2, and 3, and agrees with items 4 and 5.

ENTER ACROSS

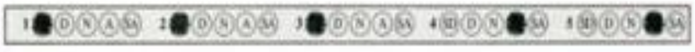

Fill in only one response for each statement. Respond to all of the statements, making sure that you fill in the correct response. **DO NOT ERASE**! If you need to change an answer, make an "**X**" through the incorrect response and then fill in the correct response.

Before responding to the statements, turn to the inside of this Item Booklet and enter your name, age, sex, ID number (if any), and today's date.

PAR • 16204 N. Florida Ave. • Lutz, FL 33549 • 1.800.331.8378 • www.parinc.com

Copyright © 1978, 1985, 1989, 1991, 2003, 2010 by PAR. All rights reserved. May not be reproduced in whole or in part in any form or by any means without written permission of PAR. This form is printed in purple and black ink on carbonless paper. Any other version is unauthorized.

987654 Reorder #RO-6806 Printed in the U.S.A.

WARNING! PHOTOCOPYING OR DUPLICATION OF THIS FORM WITHOUT PERMISSION IS A VIOLATION OF COPYRIGHT LAWS.

Name ______________________ Age _____ Sex _________ Today's date _________

1. I am not a worrier.
2. I like to have a lot of people around me.
3. I enjoy concentrating on a fantasy or daydream and exploring all its possibilities, letting it grow and develop.
4. I try to be courteous to everyone I meet.
5. I keep my belongings neat and clean.

6. At times I have felt bitter and resentful.
7. I laugh easily.
8. I think it's interesting to learn and develop new hobbies.
9. At times I bully or flatter people into doing what I want them to.
10. I'm pretty good about pacing myself so as to get things done on time.

11. When I'm under a great deal of stress, sometimes I feel like I'm going to pieces.
12. I prefer jobs that let me work alone without being bothered by other people.
13. I am intrigued by the patterns I find in art and nature.
14. Some people think I'm selfish and egotistical.
15. I often come into situations without being fully prepared.

16. I rarely feel lonely or blue.
17. I really enjoy talking to people.
18. I believe letting students hear controversial speakers can only confuse and mislead them.
19. If someone starts a fight, I'm ready to fight back.
20. I try to perform all the tasks assigned to me conscientiously.

21. I often feel tense and jittery.
22. I like to be where the action is.
23. Poetry has little or no effect on me.
24. I'm better than most people, and I know it.
25. I have a clear set of goals and work toward them in an orderly fashion.

26. Sometimes I feel completely worthless.
27. I shy away from crowds of people.
28. I would have difficulty just letting my mind wander without control or guidance.
29. When I've been insulted, I just try to forgive and forget.
30. I waste a lot of time before settling down to work.

31. I rarely feel fearful or anxious.
32. I often feel as if I'm bursting with energy.
33. I seldom notice the moods or feelings that different environments produce.
34. I tend to assume the best about people.
35. I work hard to accomplish my goals.

36. I often get angry at the way people treat me.
37. I am a cheerful, high-spirited person.
38. I experience a wide range of emotions or feelings.
39. Some people think of me as cold and calculating.
40. When I make a commitment, I can always be counted on to follow through.

41. Too often, when things go wrong, I get discouraged and feel like giving up.
42. I don't get much pleasure from chatting with people.
43. Sometimes when I am reading poetry or looking at a work of art, I feel a chill or wave of excitement.
44. I have no sympathy for beggars.
45. Sometimes I'm not as dependable or reliable as I should be.

46. I am seldom sad or depressed.
47. My life is fast-paced.
48. I have little interest in speculating on the nature of the universe or the human condition.
49. I generally try to be thoughtful and considerate.
50. I am a productive person who always gets the job done.

51. I often feel helpless and want someone else to solve my problems.
52. I am a very active person.
53. I have a lot of intellectual curiosity.
54. If I don't like people, I let them know it.
55. I never seem to be able to get organized.

56. At times I have been so ashamed I just wanted to hide.
57. I would rather go my own way than be a leader of others.
58. I often enjoy playing with theories or abstract ideas.
59. If necessary, I am willing to manipulate people to get what I want.
60. I strive for excellence in everything I do.

Enter your responses here—remember to enter responses <u>*ACROSS*</u> the rows.

SD = *Strongly Disagree*; D = *Disagree*; N = *Neutral*; A = *Agree*; SA = *Strongly Agree*

[illegible]

1 (SD) (D) (N) (A) (SA)	2 (SD) (D) (N) (A) (SA)	3 (SD) (D) (N) (A) (SA)	4 (SD) (D) (N) (A) (SA)	5 (SD) (D) (N) (A) (SA)
6 (SD) (D) (N) (A) (SA)	7 (SD) (D) (N) (A) (SA)	8 (SD) (D) (N) (A) (SA)	9 (SD) (D) (N) (A) (SA)	10 (SD) (D) (N) (A) (SA)
11 (SD) (D) (N) (A) (SA)	12 (SD) (D) (N) (A) (SA)	13 (SD) (D) (N) (A) (SA)	14 (SD) (D) (N) (A) (SA)	15 (SD) (D) (N) (A) (SA)
16 (SD) (D) (N) (A) (SA)	17 (SD) (D) (N) (A) (SA)	18 (SD) (D) (N) (A) (SA)	19 (SD) (D) (N) (A) (SA)	20 (SD) (D) (N) (A) (SA)
21 (SD) (D) (N) (A) (SA)	22 (SD) (D) (N) (A) (SA)	23 (SD) (D) (N) (A) (SA)	24 (SD) (D) (N) (A) (SA)	25 (SD) (D) (N) (A) (SA)
26 (SD) (D) (N) (A) (SA)	27 (SD) (D) (N) (A) (SA)	28 (SD) (D) (N) (A) (SA)	29 (SD) (D) (N) (A) (SA)	30 (SD) (D) (N) (A) (SA)
31 (SD) (D) (N) (A) (SA)	32 (SD) (D) (N) (A) (SA)	33 (SD) (D) (N) (A) (SA)	34 (SD) (D) (N) (A) (SA)	35 (SD) (D) (N) (A) (SA)
36 (SD) (D) (N) (A) (SA)	37 (SD) (D) (N) (A) (SA)	38 (SD) (D) (N) (A) (SA)	39 (SD) (D) (N) (A) (SA)	40 (SD) (D) (N) (A) (SA)
41 (SD) (D) (N) (A) (SA)	42 (SD) (D) (N) (A) (SA)	43 (SD) (D) (N) (A) (SA)	44 (SD) (D) (N) (A) (SA)	45 (SD) (D) (N) (A) (SA)
46 (SD) (D) (N) (A) (SA)	47 (SD) (D) (N) (A) (SA)	48 (SD) (D) (N) (A) (SA)	49 (SD) (D) (N) (A) (SA)	50 (SD) (D) (N) (A) (SA)
51 (SD) (D) (N) (A) (SA)	52 (SD) (D) (N) (A) (SA)	53 (SD) (D) (N) (A) (SA)	54 (SD) (D) (N) (A) (SA)	55 (SD) (D) (N) (A) (SA)
56 (SD) (D) (N) (A) (SA)	57 (SD) (D) (N) (A) (SA)	58 (SD) (D) (N) (A) (SA)	59 (SD) (D) (N) (A) (SA)	60 (SD) (D) (N) (A) (SA)

Now answer the three questions labeled A, B, and C below.

A. Have you responded to all of the statements? ____ Yes ____ No

B. Have you entered your responses across the rows? ____ Yes ____ No

C. Have you responded accurately and honestly? ____ Yes ____ No
